# Pre‐empting the challenges faced in adolescence: A systematic literature review of effects of psychosocial interventions for preteens with type 1 diabetes

**DOI:** 10.1002/edm2.120

**Published:** 2020-03-03

**Authors:** Elena Rey Velasco, Regitze Anne Saurbrey Pals, Timothy Charles Skinner, Dan Grabowski

**Affiliations:** ^1^ Steno Diabetes Center Copenhagen Gentofte Denmark; ^2^ Department of Psychology University of Copenhagen København K Denmark

**Keywords:** children and adolescents, education, psychological aspects, self‐management, sociological aspects

## Abstract

**Introduction:**

Numerous psychosocial interventions have been conducted in children and adolescents with type 1 diabetes, aiming to improve their self‐management and autonomy acquisition. However, these tend to address family conflict and parental perspectives, and a scarce number of interventions explore the outcomes among preteens. This review examined the outcomes of psychosocial interventions for preteens with type 1 diabetes, as an under‐researched field to date.

**Methods:**

A systematic literature review of intervention studies with randomized controlled trial design, targeting preteens with type 1 diabetes, was conducted. Six databases were searched for publication periods from 1995 to October 2019. Quality of the interventions according to the International Society for Pediatric and Adolescent Diabetes (ISPAD), as well as reporting and effect sizes, were assessed.

**Results:**

Twelve studies were selected, covering ten interventions. According to the topics identified, four of these interventions were categorized as self‐care programmes, three as psychosocial programmes and three as mixed. All of the interventions, except for one, covered ≥50% of the ISPAD recommendations. Reporting adequacy was negative only in one intervention. Main outcomes were glycemic control and self‐management, but effect sizes could only be calculated for half of the interventions with no overall significant effect.

**Conclusions:**

This review shows a lack of adequate psychosocial interventions targeting preteens with type 1 diabetes and actively involving them as participants. These intervention's educational programmes and methods should be standardized to guarantee successful results. New technologies and peer support implementation could be a promising pathway when designing these studies.

## Graphical abstract

Twelve studies were selected for this systematic review, covering ten interventions. According to the topics identified, four of these interventions were categorized as self‐care programmes, three as psychosocial programmes and three as mixed programmes combining competencies from both self‐care and psychosocial programmes. The main outcomes were glycemic control and self‐management.

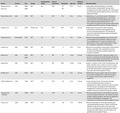

## 1 INTRODUCTION

Prevalence of Type 1 Diabetes (T1D) has alarmingly increased worldwide during recent years,^1^ with the highest rates registered among children in Finland and Sweden (57.6 and 43.1 per 100 000 per year for children aged under 15 years old, respectively).^2^ Left untreated, persistently elevated blood glucose levels are associated with long‐term complications such as clinical retinopathy, nephropathy, neuropathy and vascular disease.^3^ Achieving optimal diabetes outcomes may become challenging during different stages of life. Adolescence represents a critical period for children with T1D, in that it is associated with an increased likelihood of experiencing higher blood glucose levels and recurrent hypoglycaemia and ketoacidosis episodes, which burden adolescents' burgeoning autonomy and diabetes self‐management.^4^, ^5^ Numerous interventions have been conducted aiming to address these problems with limited success in terms of clinical and psychosocial outcomes.^6^, ^7^, ^8^ This inefficacy has been suggested to be related to the timing of these interventions, usually during adolescence,^9^, ^10^ and their tendency to target family conflict and parental perspectives. Some challenges around engaging adolescents during an intervention involve the limitations when taking the theory to practice, the difficulties with adherence and follow‐up and the use of the right methods tailored to this life period.^11^ Streisand and Mednick^10^ discuss the potential positive effects psychosocial interventions can have on self‐management behaviours and metabolic control during adolescence when interventions are administered starting in the preteenage period. Family interactions play an essential role in diabetes management among preteens and may influence self‐care and glycemic control during this developmental period.^12^, ^13^, ^14^ Likewise, a broad range of studies have reported changes in parental stress and quality of life when a child is diagnosed with diabetes.^13^, ^14^, ^15^ However, less research can be found evaluating this impact among preteens.^16^ Preteens are a term that includes children within the life‐span of 9‐12 years old.^17^

For this reason, the present systematic review examines the outcomes of psychosocial interventions for preteens with T1D, the aim being to inform the intervention research on preteens with type 1 diabetes. A systematic literature review was conducted followed by an analysis of the selected studies' results.

## 2 METHODS

To assess the results of interventions conducted among preteens with T1D, a systematic literature review was conducted. This review protocol was not preregistered. Psychosocial interventions included teaching diabetes‐related knowledge or skill, psychosocial training or support as well as psychotherapeutic interventions targeting individuals and families.^7^ Psychosocial aspects covered behavioural, psychological and social issues in relation to living with diabetes, for example self‐management, coping and communication.

### 2.1 Search strategy

A literature search was conducted in six databases: PubMed, PsycINFO, CINAHL, Web of Science, SCOPUS and Sociological Abstracts, applying a criterion for publication periods from 1995 to October 2019. This time range was based on previous systematic reviews in the field.^9^, ^18^ Two searches were conducted for each database using Medical Subject Headings (MeSH) terms and Boolean operators, being ‘Diabetes Mellitus, Type 1’ AND ‘Child’ AND (‘Self Care’ OR ‘Self‐Management’) for the first search, and ‘Diabetes Mellitus, Type 1’ AND ‘Child’ AND (‘Psychology’ OR ‘Sociology’ OR ‘Anthropology’) for the second search. These are very broad terms used to ensure that the search captured the right age span as well as an extensive range of psychosocial issues, for example mental health and quality of life.

All of the articles found were imported to EndNote and classified by database. The results obtained were compared among databases in order to discard duplicates.

### 2.2 Inclusion and exclusion criteria

No restrictions on article language were applied. The following inclusion criteria were applied: (a) type 1 diabetes, (b) age span of 7‐13, (c) involvement of preteens as intervention participants, (d) any kind of psychosocial aspect, (e) published from 1995 to 2019, (f) intervention study and (g) randomized‐control trial (RCT). Studies not involving preteen's participation and/or based on types of diabetes other than type 1 as well as other conditions were excluded. Articles that did not report any research data or only reported nonpsychosocial outcomes for the review were excluded (such as protocols or feasibility and acceptability studies).

### 2.3 Screening process

The screening process consisted of three stages: (a) a first screening for title and abstract was carried out by one coder applying criteria 1‐5. If it was unclear from the abstract whether papers met the inclusion criteria, full paper manuscripts were obtained; (b) a second screening was conducted by two coders applying criteria 1‐7, and new articles were identified by reviewing reference lists. When the studies did not meet the criteria or when this was unclear, a comprehensive review was performed; (c) full texts belonging to the remaining articles were extracted and assessed for elegibility and final inclusion for qualitative and quantitative analysis. At each step of the screening process, uncertainty about studies was discussed with the research team. Further confusion or disagreement about the studies was resolved with the research team. The study design and intervention details of the selected articles can be found in Tables 1 and 2, respectively.

**Table 1:** Study design details

Author	Country	Year	Design	Randomization details	Type of programme	Population	Age span	Diabetes duration	Brief description
Ambrosino et al	USA	2008	RCT	Yes	PSP	87	8‐12	≥6 mo	Coping skills training addressing, for example communication, social problem‐solving, stress management and conflict resolution. The intervention was informed by Social Cognitive Theory and a Stress Adaptation Model
Grey et al		2009				82			
Fiallo‐Scharer et al	USA	2019	RCT	Yes	SCP	214	8‐16	12 mo	Identification of one of three barriers (Motivation, Understanding and Organizing Care, and Family Interactions) for each family followed by a group session addressing the identified barrier. The intervention was guided by Motivational Interviewing and Behavioural Family Systems Therapy Approach
Gregory et al	UK	2011	Cluster trial	No	PSP	693	4‐15	>12 mo	Multifaceted communication skills training for professionals including a shared agenda‐setting tool and a learning programme. The training was inspired by Motivational Interviewing and Cognitive Behavioural Therapy
Henkemans et al	Netherlands	2017	RCT	No	SCP	27	7‐14	≥6 mo	Personal robot playing a diabetes quiz with the child containing general and diabetes‐related questions. The development of the robot was based on Self‐Determination Theory, addressing children's needs for competence, relatedness and autonomy
Lasecki et al	USA	2008	RCT	Yes	SCP	4	8‐12	4‐6 y	Behavioural consultations using positive reinforcement (mystery motivator). The consultations focused on problem identification, problem analysis and treatment evaluation
Nansel et al	USA	2007	RCT	Yes	MP	81	11‐16	≥12 mo	A ‘diabetes personal trainer’ supporting children and parents in reviewing self‐monitoring data, identifying areas for improvement and goal setting. The approach was informed by Motivational Interviewing
		2009							
Nansel et al	USA	2012	RCT	Yes	MP	390	9‐14,9	≥3 mo	Support in relation to clinic visits including phone calls before and after visits, as well as in‐person contact during visits. Intervention contacts were structured by the WE‐CAN problem‐solving approach, which was inspired by Social Cognitive Theory
Pendley et al	USA	2002	RCT	Yes	PSP	68	8‐17	≥15 mo	Identification of a peer support team by the child of at least three individuals from daily life including, for example school teachers, family, friends or neighbours
Sullivan‐Bolyai et al	USA	2016	RCT	No	MP	22	9‐12	≥12 mo	A teen educator mentor and a parent educator mentor facilitating education sessions on hypoglycaemia, problem‐solving and communication. The intervention was guided by the Family Style Management Framework focusing on self‐efficacy, problem‐solving and collaborative decision making
Streisand and Mednick	USA	2006	RCT	No	SCP	64	9‐11	≥6 mo	Education sessions based on the DECIDE programme as an intervention framework. The sessions included problem‐solving strategies, role play and negotiation of parent‐child diabetes management. The intervention was informed by Social Cognitive Theory
Toscos et al	USA	2012	RCT	No	SCP	48	5‐11	≥12 mo	Wireless technology for retrieving, analysing and reporting blood glucose data. Families could decide whether to take action based on the data using self‐care knowledge gained through glucose pattern management skills training. The intervention was guided by Social Cognitive Theory

Abbreviations: CST, coping skills training; DECIDE, diabetes education, counselling and information delivery, and evaluation; FMSF, family management style framework;; MP, mixed programme; PSP, psychosocial programme; RCT, randomized controlled trial; SCP:self‐care programme; SCT, social cognitive theory; SDT, self‐determination theory; UK, United Kingdom; USA, United States of America; WE‐CAN, working together, exploring barriers, choosing solutions, acting on our plan and noting solutions.

**Table 2:** Intervention details

Author	No. of sessions	Session duration	Follow‐up	Intervention duration	Delivery mode	Setting	Deliverer	Comparison group	Outcomes	Results	Other findings
Ambrosino et al (2008)	6	90 min	1, 3, 6 and 12 mo after	12 mo	Group	Clinic	Mental health professional	Controlled intervention	HbA1c, QoL, intervention acceptance/satisfaction	↓HbA1c, established relationship between self‐management behaviours and transfer of autonomy from parents to child, ↑QoL, +acceptance/satisfaction	Comparison intervention group (CST) did not have a differential effect on HbA1c or any of the child's psychosocial outcome compared to control at 3 mo, better trend towards greater improvement in life satisfaction with CST vs control, whose scores declined (*P* = .07; ES = 0.19), improvement in nearly all psychosocial outcome measures, especially self‐efficacy for diabetes management
Grey et al (2009)									HbA1c, self‐efficacy, coping skills, QoL, psychological distress	↓HbA1c, ↑self‐efficacy, = coping skills, ↑QoL, ↓psychological distress (both groups)	Children on insulin pump had lower HbA1c over time. But no significant effect differences between intervention and control groups. Socioeconomic status (medium‐to‐high) and the use of insulin pump in the majority of the subjects might have influenced the results
Fiallo‐Scharer at al. (2019)	4	75 min	12 mo after	9 mo	Group	Clinic	Interdisciplinary team	Standard care	HbA1c, QoL	↓HbA1c, =QoL	No effect on younger participants (8‐12 y old), better results at one of the clinical sites due to a significant difference on baseline HbA1c
Gregory et al (2011)	3	20 min	12 mo after	12 mo	Individual	Clinic	Interdisciplinary team	Standard care	HbA1c, QoL, psychological distress, healthcare costs	↑HbA1c, ↓QoL, ↑psychological distress (loss of confidence), =healthcare costs	
Henkemans et al (2017)	3	40‐50 min	After every session	18 wk	Individual	Clinic	Personal or neutral robot	2 intervention groups vs. standard care	Self‐management and adherence, diabetes knowledge, QoL, intervention acceptance/satisfaction	=Self‐management and adherence, ↑diabetes knowledge, ?QoL, +acceptance/satisfaction	Personal robot (intervention 1) provided more pleasure and motivation, but no other different effects with one or the other robot. Younger children were more involved
Lasecki et al (2008)	3	N/A	1 mo after	N/A	Individual	Clinic and/or school	Consultant and consultee	Controlled intervention	Blood glucose, self‐management and adherence, intervention acceptance/satisfaction	↓Mean blood glucose, ↑self‐management and adherence, +acceptance/satisfaction	Higher acceptability for consultant and consultees than for the child participants
Nansel et al (2007, 2009)	6	N/A	Post‐intervention, 6 and 12 mo after	2 mo	Individual	Home or public location	Trained nonprofessionals (students in health‐related fields)	Standard care	HbA1c, self‐management and adherence, self‐efficacy, QoL, intervention acceptance/satisfaction	↓HbA1c, =self‐management and adherence, =self‐efficacy, =QoL, +acceptance/satisfaction (higher in parents than children)	Intervention effect occurred specifically among middle adolescents and not among pre‐/early adolescents. No differences between groups at short‐term follow‐up. At 12 mo follow‐up, intervention group reported lower positive outcome expectations and higher diabetes impact
			24 mo after						HbA1c, self‐efficacy, QoL	↓HbA1c, =self‐efficacy, =QoL	
Nansel et al (2012)	6‐8	30 min	2 and 6 wk after	24 mo	Individual	Clinic	Specially trained personnel (health advisors)	Standard care	HbA1c, self‐management and adherence	↓HbA1c, =self‐management and adherence	No effect among younger patients. Intervention effects started after 12 mo (3‐4 sessions) and increased across time
Pendley et al (2002)	5	N/A	N/A	Unfinished	Individual	Home	Project coordinator or a trained under‐graduate level research assistant	Standard care	Self‐management and adherence, diabetes knowledge	N/A	Positive correlation between metabolic control and adherence, and between peer support and diabetes knowledge
Sullivan‐Bolyai et al (2016)	1	60‐90 min	2 wk after	Feasibility	Individual and group	Clinic	Teen mentor and nurse educator	Controlled intervention	Self‐management and adherence, diabetes knowledge	=Self‐management and adherence, ↑diabetes knowledge	
Streisand and Mednick 2006	3	30min‐2 h	2 wk and 1, 6, 12 and 24 mo after	Unfinished	Individual and group	Clinic	Interdisciplinary team	Standard care	Blood glucose, QoL, acceptance/satisfaction	+acceptance/satisfaction	
Toscos et al (2012)	N/A	N/A	3, 6, 9, and 12 mo after	12 mo	Individual	Clinic	Interdisciplinary team	Standard care	HbA1c, self‐management and adherence	↓HbA1c, =self‐management and adherence	

Abbreviation: QoL, quality of life.

### 2.4 Risk of bias assessment

Each of the interventions was assessed for risk of bias according to the Cochrane Collaboration's tool.^19^ This assessment consists of the following seven criteria: random sequence generation (selection bias), allocation concealment (selection bias), blinding of participants and researchers (performance bias), blinding of outcome assessment (detection bias), incomplete outcome assessment (attrition bias), selective reporting (reporting bias) and other bias due to problems not covered within the previous criteria. Each of these criteria is judged as low risk of bias, if the bias is unlikely to alter the results seriously, unclear risk of bias if this bias raises some doubt about the results and high risk of bias if the bias may alter the results seriously (Table 3).

**Table 3:** Risk of bias assessment

	Random sequence generation	Allocation concealment	Blinding of participants and researchers	Blinding of outcome assessment	Incomplete outcome data	Selective reporting	Other bias
Ambrosino et al (2008) Grey et al (2009)	−	?	?	?	−	−	+
Fiallo‐Scharer et al (2019)	−	?	+	?	−	+	−
Gregory et al (2011)	?	+	+	+	−	−	−
Henkemans et al (2017)	?	?	?	?	−	+	+
Lasecki et al (2008)	?	?	?	+	+	−	+
Nansel et al (2007, 2009)	−	−	−	+	+	−	−
Nansel et al (2012)	−	−	?	−	−	−	−
Pendley et al (2002)	+	?	?	?	?	?	+
Streisand and Mednick (2006)	?	?	+	+	?	+	?
Sullivan‐Bolyai et al (2016)	?	?	?	?	−	−	−
Toscos et al (2012)	?	?	+	+	+	−	−

Note

−: low risk of bias; ?: unclear risk of bias; **+**: high risk of bias.

### 2.5 Primary quality assessment

The quality of the educational interventions was assessed using the International Society for Pediatric and Adolescent Diabetes (ISPAD) recommendations, according to the 19 key criteria selected by Colson et al.^20^ These recommendations evaluate the impact of educational interventions on both diabetes knowledge and psychosocial outcomes such as self‐management, and belong to the ISPAD Guidelines from 2009^21^ and 2014,^22^ subsequently updated with the new recommendations published in 2018.^23^ The 19 key criteria were organized into three categories: (a) general recommendations, containing seven criteria, (b) universal principles, containing five criteria and (c) characteristics of a structured education programme, containing seven criteria. Equal weight was given to each criterion.

Every intervention was assigned a global score out of 19 based on the number of recommendations met, followed by a second score by category identifying the weak points (Table 4). A second score <50% indicates the need to improve the educational programme in that category in relation to the ISPAD guidelines.^20^ The quality assessment was conducted by one coder and checked by three other coders.

**Table 4:** Matching with ISPAD Guidelines

	Type of programme										
	Self‐care (SCP)					Psychosocial (PSP)			Mixed (MP)		
	Streisand and Mednick (2006)	Lasecki et al (2008)	Toscos et al (2012)	Henkemans et al (2017)	Fiallo‐Scharer et al (2019)	Pendley et al (2002)	Nansel et al (2007, 2009)	Ambrosino et al (2008), Grey et al (2009)	Gregory et al (2011)	Nansel et al (2012)	Sullivan‐Bolyai et al (2016)
1. General recommendations											
Based on clear theoretical psychoeducational principles	X	X		X	X		X	X	X	X	X
Integrated into routine clinical care	X		X		X				X	X	
Referred to as an ongoing process of provision of individualized self‐management and psychosocial support	X	X	X	X	X	X	X	X	X	X	X
Involves the continuing responsibility of parents and other carers throughout adolescence	X	X	X		X	X	X	X	X	X	X
Makes use of cognitive behavioural techniques most often related to problem‐solving, goal setting, communication skills, motivational interviewing, family‐conflict resolution, coping skills and stress management	X	X	X	X	X	X	X	X	X	X	X
Uses new technologies in diabetes care as one of the vehicles for educational motivation			X	X							
Delivered by an interdisciplinary team of paediatric health care professionals	X		X		X				X		
2. Universal principles											
Every young person has a right to comprehensive expert structured education	X			X	X			X		X	
Easy access for children and adolescents, both parents and other care providers	X	X	X	X	X	X	X	X	X	X	X
Diabetes education adaptable and personalized	X	X			X	X	X	X	X	X	X
Assessment of the person's attitudes, beliefs, learning style, ability and readiness to learn, existing knowledge and goals	X	X	X	X	X	X	X	X		X	X
Continuous process and repeated for it to be effective		X	X		X	X	X	X	X	X	X
3. Characteristics of a structured education programme											
It has structured, predetermined, written and evaluated curriculum	X	X	X	X	X		X	X	X	X	X
It uses trained educators	X	X			X	X	X	X	X	X	X
It is quality assured					X			X	X	X	X
It is audited					X			X		X	
It is run at a location accessible to individuals and families, whether in an ambulatory setting or not	X	X	X	X	X	X	X	X	X	X	X
It uses a variety of teaching techniques, adapted to meet the different needs, personal choices, and learning styles of youths with diabetes and their parents					X		X	X		X	X
It is enhanced by peer groups or school friendships						X		X			X
Global score (n = 19)	13	11	11	9	17	10	12	16	13	16	14

### 2.6 Reporting assessment

Reporting was assessed based on the four elements checklist for quality assessment elaborated by Carroll et al^24^ as this review started out including both qualitative and quantitative studies and was then reduced to only RCTs. Each publication was reviewed to determine whether the question and study design, participants' recruitment and selection, and the methods of data collection and analysis were reported adequately. Subsequently, the studies were dichotomized as adequately reported if they received a *Yes* on two or more criteria, and as inadequately reported if they were assigned a *Yes* on one or fewer criteria.

### 2.7 Effect sizes

Effect sizes were calculated as the standardized mean difference (SMD) between control and intervention groups for selected outcomes. These, together with the *P* values, are shown only for the interventions which provided enough information to allow for these calculations (Table 5). As suggested by GRADE, a cut‐off point of 0.5 for SMD was used as a rule of thumb for an important effect size difference.^25^

**Table 5:** Effect sizes

Intervention	Outcome		Post‐intervention	*P* value	6 mo	*P* value	12 mo	*P* value	18 mo	*P* value	24 mo	*P* value
Ambrosino et al (2008), Grey et al (2009)	HbA1c		0.13 (1 mo)	.002			0.17	.001				
			0.11 (3 mo)									
	Self‐management		N/A									
Fiallo‐Schiarer et al (2019)	HbA1c (Site 1)	8‐12 y old	−0.1 (3 mo)		−0.2		−0.1	<.05				
		13‐16 y old	−0.32 (3 mo)		−0.32		−0.26					
	HbA1c (Site 2)	8‐12 y old	−0.52 (3 mo)		−0.46		−0.29	N/A				
		13‐16 y old	0.32 (3 mo)		0.26		0.16					
Gregory et al (2011)	HbA1c						0	.5				
	Self‐management		N/A									
Nansel et al (2012)	HbA1c	9‐11 y old			−0.1	.53	−0.17	.53	−0.14	.53	−0.08	.53
		12‐14 y old			−0.03	.04	−0.04	.04	−0.44	.07	−0.52	.009
	Self‐management		N/A									
Sullivan‐Bolyai et al (2016)	HbA1c		N/A									
	Self‐management	Collaboration with parents	−2.37 (2 wk)		0.37							
		Diabetes problem‐solving	−0.6 (2 wk)		0.36							
		Diabetes communication	−2 (2 wk)		0.19							
Toscos et al (2012)	HbA1c				0.31	.02	0.38	.02				
	Self‐management				0.14	.03	−0.51	.03				

## 3 RESULTS

**Figure 1:**
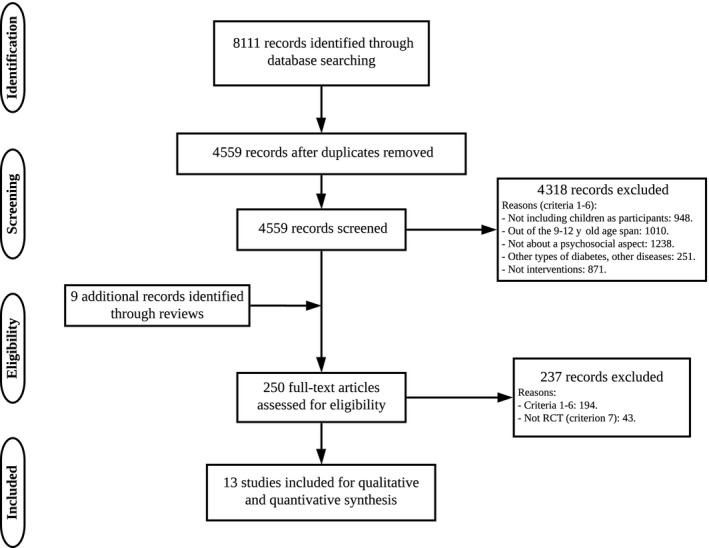
Flow chart of the screening process

The process leading to the selection of the 13 studies for the review, covering 11 interventions, can be found in Figure 1.^26^ Two articles covered results from the same intervention at different times on two occasions.^27^, ^28^, ^29^, ^30^ One of the interventions was a cluster‐randomized trial.^31^ The remaining 10 were RCTs, of which only six provided information about randomization, and coin toss was the method used in one of them.^32^ In addition, the paper by Sullivan‐Bolyai et al^17^ was a feasibility study; the authors were contacted to obtain additional information about their feasibility study and confirmed that no further data on this intervention were published after this paper.

Out of the 11 interventions, most were conducted in the United States (n = 9). The remaining two were conducted in Europe, deriving from the United Kingdom and the Netherlands. The studies were published between 2002 and 2019.

Every intervention included a comparison group, which corresponded either to a control group with standard care (n = 8) or to a different controlled intervention (n = 3). The topics identified in the studies allowed them to be classified into three categories, based on Colson et al's scheme^20^: self‐care programmes (SCP) (n = 5), psychosocial programmes (PSP) (n = 3) and mixed programmes (MP) (n = 3). The SCP included diabetes knowledge, treatment skills and self‐management, PSP covered coping, problem‐solving and communication abilities, and MP combined competencies from both SCP and PSP. The interventions included, for example coping skills training, problem‐solving and goal‐setting approaches, communication training for professionals, personal mentors, peer support and use of technology for improving diabetes knowledge and getting feedback on blood glucose monitoring (Table 1). The interventions were primarily guided by social cognitive theory and motivational interviewing.

These 11 interventions covered a total population of 1659 children between 4 and 17 years of age, always including the 7‐13 preteen age span criteria. Two of the studies analysed the results in two age groups, being 9‐11 and 12‐14 years old^33^ and 8‐12 and 13‐16, respectively.^34^ All participants had type 1 diabetes diagnosed at least 3 months,^33^ 6 months,^10^, ^29^, ^31^, ^35^ 12 months ago^17^, ^27^, ^28^, ^32^, ^34^, ^36^ or longer.^33^, ^37^

### 3.1 Delivery of the intervention

Intervention details can be found in Table 2. Seven of the interventions took place in a healthcare setting (hospital, clinic), and 1 was exclusively home‐based.^32^ Three trials gave participants the option to choose among undergoing the intervention at home, in a community setting or at a public location. Delivery modes were individual (n = 7), group (n = 2) or combined (n = 2), always including preteens as participants. Parents were included (n = 5) or free to attend (n = 6).

Four of the interventions were carried out by a multidisciplinary healthcare team. A single mental health professional conducted two of the interventions, one of which was supported by nonprofessionals belonging to the preteen's environment (ie parents, teachers). The remaining interventions were delivered by a group of trained nonprofessionals (students) (n = 2), an adolescent mentor together with a nurse advisor (n = 1), a health advisor (n = 1) and a robot (n = 1).

The number of sessions conducted varied from 1 (n = 2), 3 (n = 4), 4 (n = 1) to 5 (n = 1), 6 (n = 2), 7 and 10 (n = 1), lasting from 20 to 90 minutes. Four of the studies did not specify the session duration. Interventions lasted from 1 day (n = 1) to 18 weeks (n = 1), and 2 (n = 2), 12 (n = 4) and 24 (n = 1) months. One of them was currently ongoing on the publication date, and another one was unclear in this respect. Nine interventions performed a single (n = 4) or multiple (n = 6) follow‐ups at different intervals, between 2 weeks and 24 months post‐intervention. Henkemans et al administered questionnaires immediately after the intervention with no follow‐up,^34^ and Pendley et al^32^ did not administer any questionnaires, as it was part of Phase 1 of the intervention; the authors were contacted and confirmed that no further data were published or available for this review.

### 3.2 Outcomes and findings

#### 3.2.1 Glycemic control

Glycemic control was evaluated by measuring blood glucose in one of the interventions,^36^ and glycosylated haemoglobin (HbA1c) was the primary outcome in over half of the interventions (6 out of 11). Of these six interventions, improved blood glucose/HbA1c levels post‐intervention were shown in 5^27^, ^28^, ^29^, ^31^, ^32^, ^34^, ^36^, ^38^ and enhanced levels by a one percentage point in the remaining one.^31^ Also, three of these interventions displayed an effect in older participants only (11‐ to 13‐, 13‐ to 16‐ and 12‐ to 14‐year‐olds).^28^, ^34^, ^38^

#### 3.2.2 Self‐management and adherence

Five interventions measured diabetes treatment self‐management using the Diabetes Self‐Management Profile (DSMP),^36^, ^38^ which is a tailored version for nonprofessional interviewers,^27^ and behavioural and self‐monitoring checklists together.^36^ The DSMP includes 23 questions assessing 5 aspects of self‐management: exercise, management of hyperglycaemia, diet, blood glucose testing and insulin administration and dose adjustment. It should be administered by professionals to parents and children together when the children are <11 years old, and to the children alone when they are >11 years old.^37^ Only the study by Lasecki et al showed a slightly positive effect on self‐management. Three of the five individual subscales belonging to the Self‐Management of T1D in Adolescents (SMODA) questionnaire, containing 30 items, were used by Sullivan‐Bolyai et al to assess Self‐Management: Collaboration with Parents, Diabetes Problem Solving and Diabetes Communication; they found small and no significant differences post‐intervention.^17^

Two additional scales were used to measure self‐management and medication‐taking together. Pendley et al, and Henkemans et al and Fiallo‐Scharer et al applied two different versions of the Self‐Care Inventory (SCI) including, respectively, a 15‐item individual scale measuring the how many of the common T1D regimen tasks individuals did^39^ and 14‐items with 4 subscales corresponding to blood glucose management, insulin and food regulation, exercise and emergency precautions.^34^, ^40^ Both questionnaires can be filled in using a 5‐point Likert scale.^10^, ^33^ The Diabetes Responsibility and Conflict Scale was used by Ambrosino and colleagues^29^ to establish a relationship between self‐management behaviours and the transfer of autonomy from parents to the preteen. Henkemans et al were unclear about the existence of a positive impact on self‐management post‐intervention, and Pendley et al did not show actual scores but a positive correlation between metabolic control and self‐management. Fiallo‐Scharer et al did not report any self‐management outcomes.

#### 3.2.3 Self‐efficacy

Self‐efficacy was measured using the Self‐Efficacy for Diabetes Scale in two interventions.^27^, ^31^ This scale evaluates self‐perceptions or patients' confidence in successfully managing diabetes; it consists of 35 items assembled in 3 subscales: diabetes‐specific self‐efficacy, medical situations self‐efficacy and general situations. Nansel et al^27^ also incorporated the Outcome Expectations of Diabetes Self‐Management Positive and Negative scales, consisting of two different scales of 12 items each, to assess the strength of beliefs in positive and negative diabetes management outcomes. On the other hand, Fiallo‐Scharer et al used the Confidence in Diabetes Self‐Care (CIDS) scale to measure diabetes management self‐efficacy beliefs. This is a 20‐item self‐report questionnaire, addressed to patients with type 1 diabetes, assessing self‐efficacy as the perceived ability to perform diabetes self‐care tasks.^41^ Only the intervention by Grey et al showed benefits on self‐efficacy.

#### 3.2.4 Diabetes knowledge

Diabetes knowledge was measured by Henkemans et al using a nonvalidated 30‐item multiple‐choice questionnaire based on their previous pilot study and adapted to the training provided by their hospital and intervention, which resulted in positive learning outcomes post‐intervention. Sullivan‐Bolyai et al used a hypoglycaemia teaching‐adapted version of the Diabetes Awareness and Reasoning Test (DART), consisting of a 25‐item multiple‐choice questionnaire measuring diabetes knowledge in both preteens and parents, showing improvement in both groups, though more in the intervention group. However, differences were not considered significant due to the small number of participants (22). This intervention also applied the Wysocki's Modified Problem‐Solving Measure (PSM), consisting of five multiple‐choice items presented during a problem‐solving interview and meant to measure the problem‐solving abilities and knowledge synthesis among preteens. The accuracy of the answers improved in three out of the five problems for both groups, with higher scores for the intervention group in two of them, and lower scores on the other two problems for both groups. However, these outcomes were not significant.^17^

Pendley et al used the Diabetes Patient Knowledge Test (DPKT), which contains 23 multiple‐choice items assessing diabetes‐ and nutrition‐related knowledge. This test has only been validated in adults, but according to them, there is no other diabetes knowledge test suitable for children that can account for strong psychometric properties. Their findings showed a correlation between peer support and diabetes knowledge.^32^

#### 3.2.5 Coping skills

Coping was measured in one intervention^29^ using the Issues in Coping with T1D—Child Scale. This scale consists of 12 items inside of 2 subscales rated on a 4‐point Likert‐type scale, the aim being to assess perceptions of how hard or difficult to handle and how upsetting T1D management is. The intervention showed no differences between groups regarding this outcome.

#### 3.2.6 Quality of life

The Diabetes Quality of Life (DQOL) Scale consists of three subscales that evaluate disease impact (21 items), disease‐related worries (8 items) and diabetes life satisfaction (18 items); it was used in two interventions,^27^, ^31^ of which Ambrosino et al (3 months post‐intervention) showed an improvement on life satisfaction only, and Grey et al revealed a positive impact overall (1‐year post‐intervention). No effects were seen in Nansel et al A modification of this scale—known as The Diabetes Quality of Life Scale for Youth—was used in Ambrosino et al, the goal being to adapt it to preteen's perceptions. Other scales, such as the Pediatric Quality of Life (PedsQL) Inventory Generic Score Scales^42^ and the Health‐Related Quality of Life (HRQoL), a Dutch version of the Questionnaire for young people (DISABKIDS) with diabetes, were used by Gregory et al and Fiallo‐Scharer et al, and Henkemans et al, respectively, to measure this parameter. The DISABKIDS project was developed by the European Commission in seven European countries to standardize the instruments used to assess QoL in children with chronic diseases. These instruments correspond to questionnaires including a 37‐item generic module (DISABKIDS Chronic Generic Module, DCGM‐37) and 10‐item disease‐specific modules (DISABKIDS Diabetes‐Specific Module, DDM‐10).^43^ Results of the intervention by Gregory at al. were adverse when compared to baseline, those of the intervention by Henkemans et al were not reported and there were no changes after the intervention by Fiallo‐Scharer et al.

#### 3.2.7 Psychological distress

The Children's Depression Inventory (CDI) is a 27‐item questionnaire completed by the preteen that reflects affective, cognitive and behavioural symptoms of depression. Grey et al used the CDI, showing an improvement post‐intervention.^29^ Gregory et al used the Problem Areas in Diabetes Survey (PAID) to assess diabetes‐related psychosocial adjustment and distress,^44^ obtaining negative results that showed a loss of confidence in the preteen's ability to manage diabetes post‐intervention.^31^

#### 3.2.8 Intervention acceptance and/or satisfaction

Acceptability of the intervention was measured using study‐specific nonvalidated surveys rating the programme's participants' satisfaction and usefulness as well as the helpfulness of the deliverers through a 1 to 5,^29^, ^35^ 1 to 6^27^ or 1 to 7^34^ rating Likert scales. Two of them included open‐ended questions for commenting about likes, dislikes or suggestions. Additionally, Henkemans et al used the number of rounds the preteen decided to play to measure motivation. All of them reported positive participant feedback on the intervention. Lasecki et al^36^ used the validated Behavioral Intervention Rating Scale‐Revised (BIRS‐R) and Children's Intervention Rating Profile (CIRP) to measure the acceptability of the intervention among parents and school consultees and among preteens, respectively, showing higher scores for parents and school consultees than for preteens.

The paper by Streisand and Mednick offer a detailed description of the intervention programme followed in their ongoing randomized controlled trial. The methods section states that self‐report questionnaires and 24‐hour recall interviews were used to assess behaviour, mood and diabetes‐specific measures of QoL, parent involvement and parent‐child conflict. Blood glucose metres and medical records provided health outcome data. Besides this statement, satisfaction outcomes were reported for only 10 parents and 5 preteens (out of 64 dyads), showing positive results. Other intervention outcomes and measurement scales were not specified. The authors were contacted for further information; they confirmed that the study data were no longer available and that the final outcomes were never published.^10^

### 3.3 Risk of bias assessment

Assessment of risk of bias can be found in Table 3. This was a complicated process due to the lack of randomization details in 5 of the 11 interventions, that difficulted the assessment of *selection bias* as random sequence generation and allocation concealment criteria. Moreover, blinding was not specified or partly described in many occasions, leaving the risk of bias as an unclear statement in 6 out of the 11 interventions. The highest risk of bias (in 5 out of 11 interventions) was indeed found for the *detection bias* as blinding of outcome assessment, as researchers assessing the outcomes were frequently nonblinded. The criterion where the lowest risk of bias was found was the one regarding *reporting bias* as selective reporting, due to most of the interventions adequately reporting all the prespecified outcomes (7 out of 11). Similarly, criteria regarding both *attrition bias* as incomplete outcome data and *other bias* accounted for a low risk of bias in 6 out of 11 interventions. Drop‐out rates were low in general and some of the interventions described a method to deal with this missing data that is intention to treat analysis, and most of the bias was covered by using the Cochrane Collaboration's tool.

### 3.4 Quality of the interventions

Table 4 shows the degree of match between the Educational Programmes identified in the 11 interventions and the ISPAD Criteria. SCP showed the slightly lowest score average (12.2 out of 19) compared to PSP (12.7 out of 19) and MP (14.3 out of 19). The intervention meeting the most criteria (17 out of 19) was the one carried out by Fiallo‐Scharer et al^34^ All of the interventions covered >50% of the recommendations except one of them, which met 9 out of the 19 criteria.^34^ An analysis of the three categories stated by the ISPAD Guidelines was conducted to facilitate the identification of the strengths and weaknesses of every intervention.

#### 3.4.1 General recommendations

The main points that all the programme types lacked were the *use of new technologies* (2 out of 10) and the *presence of an interdisciplinary team delivering the intervention* (4 out of 10). The remaining recommendations were present in most of the programmes.

#### 3.4.2 Universal principles

The main reason for downgrading a point on this category was the recommendation regarding the *right to comprehensive expert, structured education for every young person*, owing to the diabetes duration criterion and other exclusion criteria such as the most recent HbA1c levels^36^, ^37^, ^38^ and being a girl with menarche.^17^ All of the programmes accomplished at least 50% of the universal principle's recommendations.

#### 3.4.3 Characteristics of a structured programme

The fewer recommendation matches found for this category were for the *audition* (3 out of 10) and *the peer groups or school friendship presence* (3 out of 10), followed by the *quality assurance* (5 out of 10) of the programme delivered and the *variety of teaching techniques used in the intervention* (5 out of 10).

### 3.5 Reporting assessment

Of the 11 interventions, all qualified as being adequately reported except for the paper by Streisand and Mednick,^10^ which did not accomplish any of the reporting questions evaluated. Regarding the remaining 10 interventions, all of them reported adequately their methods of analysis, and the main weak point was the second question, where only four of them^27^, ^28^, ^32^, ^34^, ^38^ included an explicit description of and reason for their selection of participants.

### 3.6 Effect size

A summary of effect sizes can be found in Table 5. SMD was calculated for HbA1c and self‐management outcomes immediately post‐intervention and 6, 12 and 24 months post‐intervention, when applicable, for every intervention. These effects could only be calculated for over half of the interventions (6 out of 11), of which numeric results for HbA1c and Self‐management were shown in 5 and 2 of them, respectively.

HbA1c size effects were 0.13 after 1 month, 0.11 after 3 months and 0.17 after 24 months for Ambrosino et al, and Grey et al, as well as 0.31 after 6 months and 0.38 after 12 months for Toscos et al and 0 for Gregory et al.

Self‐management size effects were 0.14 after 6 months and −0.51 after 1 year for Toscos et al and −2.81 (Collaboration with parents), −0.62 (Diabetes problem‐solving) and −2.17 (Diabetes communication) after 2 weeks for Sullivan‐Bolyai et al.

Nansel et al 2012 presented HbA1c for all the age groups together (9‐14) and separately for young (9‐11) and old (12‐14) adolescents 6, 12, 18 and 24 months post‐intervention. Effect sizes were −0.1, −0.17, −0.14 and −0.08 for young adolescents, and −0.03, −0.04, −0.44 and −0.52 for old adolescents. This was also the case of Fiallo‐Scharer et al, which presented their results separately according to the intervention site and the age of the participants as a youth (8‐12) or teen (13‐16). These were 0.1 after 3 months, 0.2 after 6 months and 0.1 after 12 months for youths, and 0.32 after 3 months, 0.32 after 6 months and 0.26 after 12 months for teens in Site 1, and 0.52 after 3 months, 0.46 after 6 months and 0.29 after 12 months for youths, and −0.32 after 3 months, −0.26 after 6 months and −0.16 after 12 months for teens in Site 2.

Regarding the remaining interventions, no numerical data were available for calculating SMD, or the data were incomplete, as in Nansel et al 2007, 2009^27^, ^28^ where SD was missing for HbA1c outcomes. Similarly, the intervention by Lasecki et al^36^ showed effect sizes for every participant without comparison between groups, and no SD was provided, meaning that these calculations could not be confirmed.

## 4 DISCUSSION

Results from this systematic literature review indicate that there have been only a few trials of psychosocial interventions targeting and actively involving preteens in the age range 9‐12 years when compared with the number of trials for adolescents/teenagers.

Comparison among interventions became complicated owing to the wide and heterogeneous variety of educational programmes, frequently with nonstandardized methods and measurement instruments, as it could be seen when assessing the risk of bias (Table 3). The classification suggested by Colson et al^20^ facilitated this process, and intervention training programmes could be identified such as the SCP (n = 5), PSP (n = 3) or MP (n = 3). The interventions showing the most pronounced results regarding HbA1c corresponded to two SCP. These were the one conducted by Toscos et al with an effect size, still moderated, of 0.38 after 12 months of intervention, and the one conducted by Fiallo‐Scharer et al with an effect size of 0.32 after 3 months of intervention exclusively in older participants (13‐16 years old). The SCP applied by Lasecki et al also reported positive results on blood glucose during treatment. However, effect sizes could not be calculated and, moreover, blood glucose levels increased 1‐month post‐intervention. It should also be noted that this intervention revealed higher acceptability as rated by the deliverer and the parents than as rated by the preteens. In contrast to this, the SCP by Henkemans et al increased diabetes knowledge and involvement among younger children, showing a promising pathway for interventions with preteens when games and new technologies are included. The remaining SCP belonged to Streisand and Mednick, but no other results than preliminary acceptance could be extracted and thereby analysed. Nonetheless, the average score matching with ISPAD Guidelines was the lowest when evaluating each type of educational programme (12.2 out of 19).

Regarding PSP, the intervention conducted by Ambrosino et al and Grey et al was the only one where effect sizes could be calculated, with very moderate results for HbA1c (0.17 effect size after 12 months). Still, nonsignificant differences between groups were found. In addition, besides of the highest risk of bias found for Gregory et al, added negative results showed a one percentage point increase in HbA1c and a loss of confidence in diabetes management, together with the failure to publish final results by Pendley et al, suggest a lower effect for this type of educational programme. Lastly, the 3 MP analysed in the present review showed the highest match with ISPAD Guidelines, the lowest risk of bias and also the best results, albeit two of them belonged to Nansel et al's research team and found no effect among younger participants, their age spans being 11‐16 and 9‐14.9, respectively. This is a similar effect as seen above with the SCP intervention by Fiallo‐Scharer et al The remaining MP educational programme showed promising results for increasing diabetes knowledge, but corresponded to feasibility results for an intervention that, to our knowledge, was never published. Thus, besides having a well‐structured programme, these findings suggest the need for educational programmes tailored to the age of the target population, especially when this may cover two life stages (pre‐adolescence and adolescence) and young people who can be divided into two groups that might benefit from receiving a specially adapted intervention. As already stated, the opposite effect was seen in Henkemans et al, when preteens expressed greater satisfaction.

Matching with ISPAD criteria revealed some critical points to be accounted for when designing an educational intervention with these characteristics. Use of new technologies, especially games, should be encouraged, as recent studies have shown positive results, particularly for self‐management behaviours such as blood monitoring.^45^ Additionally, a recent review assessing the effectiveness of peer‐based interventions targeting children and adolescents with type I diabetes confirmed its benefits, especially as a booster in circumstances where professional deliverers and an adequate educational programme are lacking.^46^ It is noteworthy that Pendley et al were the only intervention specifically focused on peer support and that the interventions of Ambrosino and Grey et al and Sullivan‐Bolyai et al included it as a variable, revealing one of the main action points when developing this kind of interventions. In fact, the lack of an interdisciplinary team including professional deliverers might be one of the reasons why most of the interventions evaluated did not succeed or incurred on a high risk of bias when reporting results inappropriately. Additionally, most of the programmes were not quality assured and/or audited or did not mention it. The universal principle regarding the *right of every young person to comprehensive expert, structured education* was not considered in the majority of interventions, as a minimum of 6 months duration of diabetes was established as a criterion for 90% of the interventions, being 4‐6 years duration of diabetes in one occasion.^36^ The argument for this criterion application was explained in Pendley et al, referring to the ‘honeymoon’ period after diagnosis, where small doses of exogenous insulin are needed as the pancreatic cells are still able to produce insulin, and thereby blood sugar changes cannot be exclusively associated with self‐management behaviours. However, diagnosis usually occurs during childhood and pre‐adolescence, so recently diagnosed children might benefit from psychosocial interventions that prepare them for the upcoming diabetes management and improve self‐efficacy outcomes in the future.^47^ Some studies have even revealed the possibility of a slowdown, aiming to stop the remission when combined with pharmacological therapies.^48^, ^49^ Other criteria, identified as interfering with the above‐mentioned universal principle, were the presence of menarche as an exclusion criterion^17^ and the most recent HbA1c% reported, the main aim being to recruit participants with poor glycemic control. Nonetheless, conducting this type of intervention in well‐regulated preteens with type 1 diabetes can take advantage of peer support, as already stated, because these preteens can learn new techniques and skills to be transmitted, through a role‐model function, to peers with diabetes who have lower metabolic control, as pointed out by Colson^20^ regarding Bandura's social learning theory.^50^

Furthermore, the country of intervention can play an important role when carrying out an educational programme. Only 2 of the 11 interventions assessed were conducted in a country other than the United States, making it difficult to generalize these results to preteens worldwide. This highlights the importance of studying preteen's needs and points of view, but also their ethnicity and race if we are to adapt the intervention to the implementation context. Likewise, socioeconomic status affects diabetes morbidity^51^ and is highly connected to the use of healthcare services,^52^ though it was only measured by one of the interventions with no significant results.^31^ Grey et al also mentioned this factor, suggesting an association between their positive results on HbA1c and the medium‐to‐high socioeconomic status of the participants.

Besides this educational programme classification and the ISPAD Guidelines, comparison problems remained when assessing the methodology used. A heterogeneous variety of questionnaires and scales were used to evaluate diverse intervention outcomes, which were common among interventions on a few occasions, but usually modified to address the target population.^10^, ^33^ The need for a standardized methodology, specifying clear measurement instruments and outcomes to assess, becomes evident when examining the results of the present review.

Along with these findings, the limited number of psychosocial interventions conducted to date among preteens with type 1 diabetes it is a matter of concern, especially those actively involving preteens as participants, one of the main exclusion criteria used in the present literature search. Likewise, preteen's needs, feelings and points of view should be considered when designing these interventions, while including family and peers. This combination would allow preteens to achieve meaningful behavioural change, along with emerging diabetes management autonomy, that will form a foundation on their way to a good metabolic control and psychosocial well‐being during adolescence and, consequently, adult life.

## CONFLICT OF INTEREST

The authors declare no conflict of interest.

## AUTHOR CONTRIBUTIONS

Rey Velasco, E. undertook the data collection, data analysis and wrote the first draft of the manuscript. Pals, RAS acted as a second reviewer for study selection and supported data analysis and the final draft of the manuscript. Skinner, T. provided crucial academic input on the search and interpretation of the data and supported data analysis and the contents of the manuscript. Grabowski, D. conceived the review, provided academic supervision, and supported data interpretation, data analysis and the contents of the manuscript.

## ETHICAL APPROVAL

Since this is a systematic review, ethical approval was not required.

## Data Availability

All relevant data are included in the manuscript.
